# Niobium(V) Oxynitride: Synthesis, Characterization, and Feasibility as Anode Material for Rechargeable Lithium-Ion Batteries

**DOI:** 10.1002/chem.201102653

**Published:** 2012-03-29

**Authors:** Xiao-Jun Wang, Frank Krumeich, Michael Wörle, Reinhard Nesper, Laurent Jantsky, Helmer Fjellvåg

**Affiliations:** [a] Dr. X.-J. Wang, Dr. F. Krumeich, Dr. M. Wörle, Prof. Dr. R. Nesper Laboratory of Inorganic Chemistry, ETH ZürichWolfgang-Pauli-Strasse 10, 8093 Zürich (Switzerland), Fax: (+41) 44-632-1149; [b] Dr. L. Jantsky, Prof. Dr. H. Fjellvåg Department of Chemistry, Centre for Materials Science and NanotechnologyUniversity of Oslo, FERMiO, 0349 Oslo (Norway)

**Keywords:** electrochemistry, nanoparticles, niobium, oxynitrides, solid-state reactions

## Abstract

**Abstract:**

The decomposition reaction of niobium(V) oxytrichloride ammoniate to the oxynitride of niobium in the 5+ oxidation state was developed in a methodological way. By combining elemental analysis, Rietveld refinements of X-ray and neutron diffraction data, SEM and TEM, the sample compound was identified as approximately 5 nm-diameter particles of NbO_1.3(1)_N_0.7(1)_ crystallizing with baddeleyite-type structure. The thermal stability of this compound was studied in detail by thermogravimetric/differential thermal analysis and temperature-dependent X-ray diffraction. Moreover, the electrochemical uptake and release by the galvanostatic cycling method of pure and carbon-coated NbO_1.3(1)_N_0.7(1)_ versus lithium was investigated as an example of an Li-free transition-metal oxynitride. The results showed that reversible capacities as high as 250 and 80 A h kg^−1^ can be reached in voltage ranges of 0.05–3 and 1–3 V, respectively. Furthermore, a plausible mechanism for the charge–discharge reaction is proposed.

## Introduction

Nowadays, it is a consensus that the rechargeable Li-ion battery is one of the best solutions for energy storage. Even though intensive research on Li-ion batteries has been carried out in recent decades, the development of electrode materials with high energy densities still remains a big challenge.^1^ One large family of compounds applied in electrodes for Li-ion batteries are transition-metal oxides. This includes not only materials for cathodes, such as LiMO_2_ (M=Mn, Co, and Ni), but also for anodes, like Fe_3_O_4_, CuO, and others.^2^ Another type of electrode materials is comprised of transition-metal nitrides, which include lithium insertion compounds, for example, Li_3_FeN_2_, Li_3−*x*_M_*x*_N (M=Co, Ni, Cu), and Li_7_MnN_4_, as well as lithium-free compounds like CoN, Cr_1−*x*_Fe_*x*_N.^3^ Usually, nitrides have low work potentials because of the lower electronegativity of nitrogen compared to oxygen as well as the associated lower oxidation states of the transition metals. However, recently it was realized that, in carbonate-based electrolyte batteries, anodes with a very low work potential (<1 V) may destroy the solid electrolyte interface (SEI) and thereby trigger short-circuits and electrolyte ignition during fast charge. These unwanted properties provide a strong motivation to search for new substitutes for graphite- and nitride-based anodes.^1^ In the development of safer and longer-lived batteries, TiO_2_ and Li_4_Ti_5_O_12_ attracted some attention and thus were investigated intensively.^4^ Li_4_Ti_5_O_12_ shows a practical capacity as high as 200 A h kg^−1^ and a proper potential plateau at 1.5 V versus Li^+^/Li^0^ resulting from the Ti^4+^/Ti^3+^ redox couple.^2^ Also, the Nb^5+^/Nb^4+^ couple has a potential around 1.5 V versus Li^+^/Li^0^ in oxoniobates, and reduction to the Nb^4+^/Nb^3+^ couple may further increase the lithiation capacity of such compounds. For example, Nb_2_O_5_ and various niobates like AlNbO_4_, KNb_5_O_13_, and K_6_Nb_10.8_O_30_ exhibit outstanding electrochemical properties as anodes of Li-ion batteries.^5^

We believe that transition-metal oxynitrides are promising candidates for both anode and cathode applications, too. Recent studies started to investigate transition-metal oxynitrides in various applications, such as ionic conductivity, catalysis, pigments, and thermoelectrics.^6^ However, only a few of them were investigated as electrode candidates for Li-ion batteries. The first transition-metal oxynitride used as electrode material in Li-ion batteries was Li_7.9_MnN_3.2_O_1.6_, which exhibited a similar electrochemical behavior to Li_7_MnN_4_ but showed improved chemical stability.^7^ In principle, transition-metal oxynitrides have higher theoretical capacity than the corresponding oxides because of their higher lithiation capacity per unit weight due the higher charge of nitride compared to oxide anions. Unfortunately, the number of known transition-metal oxynitrides is quite limited due to the restrictions of classical ceramic sintering synthesis methods for nitridation and the difficulty of determining N/O ratios. Up to now, the electrochemical investigation of transition-metal oxynitrides has mostly been focused on metals of groups 4–6.^8^ To investigate the feasibility of oxyntrides as electrode materials for Li-ion batteries, ternary oxynitrides with lighter transition metals in high oxidation states were preferentially selected, because electrode materials are required to have a high specific capacity. Additionally, proper redox couples are necessary.

**Figure 1:**
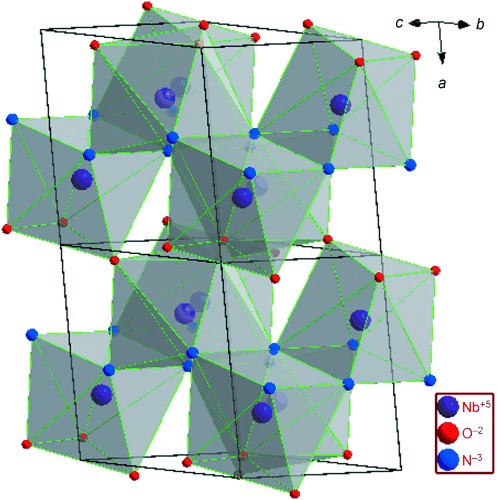
Crystal structure of NbON.

TaON was the first ternary oxynitride with a full oxidized state obtained by the ammonolysis method. It was first reported by Brauer et al. in 1966 and recently studied as a new pigment candidate.^9^ Oxynitrides of vanadium in the 5+ oxidation state can be prepared by ammonolysis only when Ba is present in the structure.^10^ In contrast, NbON was not accessible through a simple ammonolysis reaction. In 1977, single crystals of NbON were grown by treating NbOCl_3_ with an excess of NH_4_Cl at 900–1000 °C and used to determine its crystal structure.^11^ Also, it was reported that black powders of NbON can be obtained by decomposition of niobium(V) oxytrichloride ammoniate.^11^ NbON and TaON are isostructural, having the baddeleyite (ZrO_2_) structure with monoclinic symmetry (space group *P*2_1_*/c*). As shown in Figure 1, Nb atoms are surrounded by three oxygen and four nitrogen atoms to form seven-coordinate polyhedra [NbO_3_N_4_], which are connected by edge-sharing N and corner-sharing O atoms. Nb^V^ oxynitride is a semiconductor due to the empty d band of niobium. NbON has a calculated band gap of 1.7 eV, which results in the intrinsic blue-colored appearance of the compound.^12^ We expected this type of compound to have the advantage of better electronic conductivity compared to pure oxides like LiFePO_4_ and most of the other commonly used electrode oxides. In the present work, we investigated reaction conditions, thermal analyses, crystal structures, and micro-characterization of the products. Pure and carbon-coated compounds were prepared to study their electrochemical performance in Li-ion batteries, and a cycling mechanism was proposed as well.

## Results and Discussion

**Figure 2:**
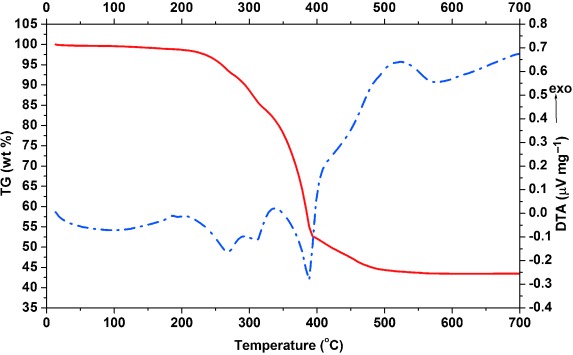
TG (—) and DTA (•–•–) curves of NbOCl_3_(NH_3_)_*x*_.

**Reaction of NbOCl_3_** **with NH_3_**: Niobium(V) oxytrichloride ammoniate NbOCl_3_(NH_3_)_*x*_ was prepared by treating NbOCl_3_ with ammonia gas at room temperature. Figure 2 shows the TG and DTA curves of NbOCl_3_(NH_3_)_*x*_ in argon atmosphere. It indicates that decomposition of NbOCl_3_(NH_3_)_*x*_ starts at 200 °C and is complete at 400 °C. Considerable heat of reaction is released. According to the weight loss during the decomposition, the molecular formula of the starting yellow niobium(V) oxytrichloride ammoniate was determined to be NbOCl_3_(NH_3_)_4_. A constant product weight is reached above 500 °C.

**Figure 3:**
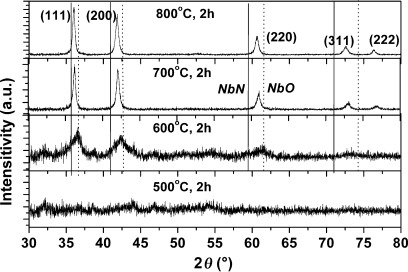
XRD patterns of products obtained by reaction between NbOCl_3_ and NH_3_ at different temperatures. — and •••• straight lines mark the positions of XRD reflections for NbN (PDF # 38-1155) and NbO (PDF # 42-1125), respectively.

As plotted in Figure 3, formation of a rock-salt phase started when the temperature reached 600 °C. Compared to XRD patterns of the isostructural compounds NbN and NbO, the observed lattice constants for products obtained at 700 and 800 °C are in between the cases of pure NbO and NbN, respectively. These compounds can be identified as niobium oxynitride NbO_*x*_N_*y*_ (0<*x*, *y*<1), which was reported previously.^13^ Obviously, increasing the temperature results in products with better crystallization and higher nitrogen concentration. The overall reaction process can be described by chemical reactions 

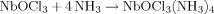
(1)


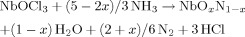
(2)

Reaction (1) is active at room temperature. When the temperature is increased to 500 °C, reaction (2) takes place.

On the basis of the TG and DTA results discussed above, the decomposition process of NbOCl_3_(NH_3_)_4_ can be formulated as Equation .

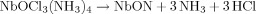
(3)

NH_4_Cl decomposes into NH_3_ and HCl above 380 °C. In a first attempt of this synthesis, dynamic vacuum and inert gas (Ar) were employed to remove NH_3_ and HCl. Surprisingly, a black-colored Nb_2_O_5_ phase, indicating oxygen deficiency and/or N doping, was obtained instead of NbON. We then changed the procedure to heating the starting material in a closed Pyrex tube and successfully produced NbON. However, overpressure results in the closed tubes from the formation of NH_3_ and HCl. To eliminate the overpressure two modifications were employed; either a long Pyrex tube in a vertical tube furnace was used so that the NH_3_ and HCl gases formed from the decomposition of NH_4_Cl at the hot bottom of tube the gases could recombine into solid NH_4_Cl on the cold top of the tube outside the furnace, or the NH_4_Cl was treated before it started to decompose. This process could be formulated as Equation .

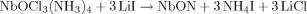
(4)

The reaction conditions used to prepare five different samples are presented in Table 1.

**Table 1:** Reaction conditions of differently prepared samples.

	molar NbOCl_3_(NH_3_)_4_/LiI	*T* [°C]	*t* [h]
**0**	1:3	500	20
**1**	1:0	500	20
**2**	1:6	500	5
**3**	1:6	500	20
**4**	1:6	500	40

**Figure 4:**
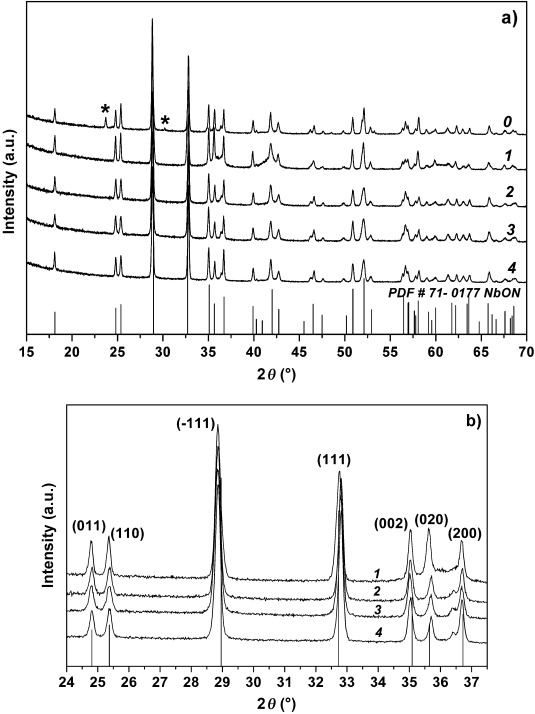
XRD patterns of samples 0–4 (see Table 1). *: The peaks of an impurity that matches with LiNbO_3_. a) Angular range from 15 to 70°. b) Angular range from 24 to 37.5°.

**Crystallographic characterization**: Figure 4 shows the XRD patterns of the five different samples. Clearly, excess LiI helps to get rid of LiNbO_3_ impurities. This could be explained in the following way: excess LiI can form eutectic mixtures with LiCl/NH_4_I and acts as a proper flux to provide homogeneous conditions for the reaction. In this case, unlike in the direct decomposition reaction of NbOCl_3_(NH_3_)_4_, little gas is released and overpressure in the reactor can be avoided. Figure 4 b zooms in on the specific diffraction-angle range of these XRD patterns. The patterns of the samples obtained here do not match the data collected in 1977.^11^ Although both samples obviously have the same monoclinic structure, different phases have been formed.

**Figure 5:**
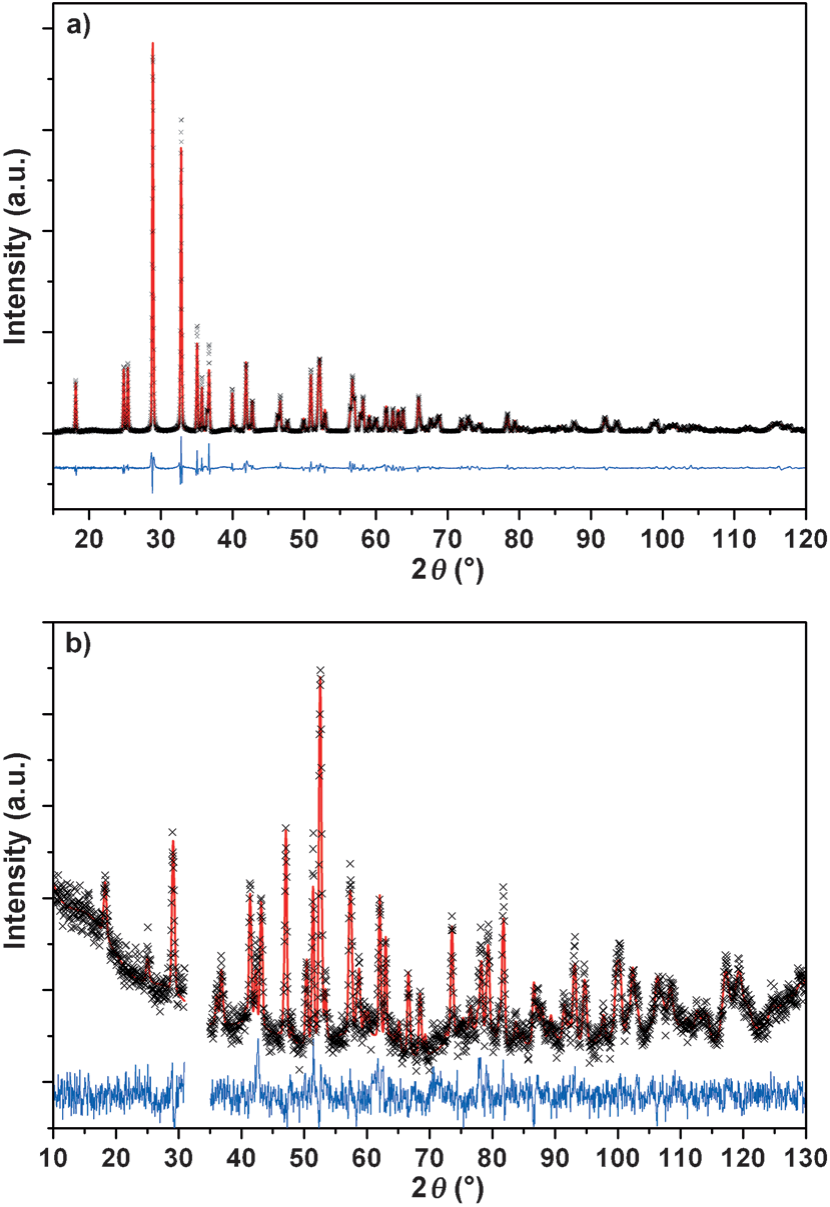
Rietveld refinement plots of NbO_1.3(1)_N_0.7(1)_ (sample 3) from a) XRD data and b) neutron diffraction data; ×: observed, —: calculated, —: difference plot.

Considering the various possible oxidation states of niobium, the oxygen and nitrogen ratio in the NbON phase obtained here is likely to be greater than 1:1 with Nb valence between +4 and +5. Elemental analysis showed samples **0** to **4** have similar N/O content, and to elucidate the crystal structure in detail, sample **3** was chosen for further investigation. The composition of final product **3** was determined as NbO_1.3(1)_N_0.7(1)_ by hot-gas N/O elemental analysis, which means that 1 of 1.3 O atoms occupy the oxygen positions in the structural model of NbON from ref. ^11^, and the nitrogen positions are occupied by 0.3 O and 0.7 N. As summarized in Table 2, Rietveld refinement of the XRD pattern (Figure 5 a) showed lattice constants slightly different from those of reported NbON, and no further information concerning the N/O ratio was found. However, in the Rietveld refinement of neutron diffraction data (Figure 5 b), when the value of N/O was fixed as 1, the displacement parameter *U*_iso_ had negative values that are physically meaningless. In contrast, refinement with unrestrained N/O content gave reasonable results: instead of full occupancy of nitrogen in NbON, the nitrogen position was occupied by 0.64(5) O and 0.36(5) N atoms, which also agreed with the results of elemental analysis.

**Table 2:** Refinement results.

	NbON^11^	3	3	3
Conditions	fixed N/O=1:1	fixed N/O=1:1	fixed N/O=1:1	refine N:O
Radiation	X-ray (Cu_Kα1/2_)	X-ray (Cu_Kα1/2_)	neutron	neutron
*λ* [Å]	1.5406	1.5406	1.5561	1.5561
*T* [K]	298	298	298	298
space group	*P*12_1_/*c*1	*P*12_1_/*c*1	*P*12_1_/*c*1	*P*12_1_/*c*1
*α* [Å]	4.970(3)	4.9770(1)	4.9808(6)	4.9808(5)
*b* [Å]	5.033(3)	5.0217(9)	5.0251(7)	5.0250(6)
*c* [Å]	5.193(3)	5.2053(1)	5.2097(7)	5.2097(6)
*β* [°]	100.23	100.754(3)	100.747(8)	100.747(7)
*V* [Å^3^	127.83	127.814	128.106(36)	128.103(34)
*Z*	4	4	4	4
Nb
*x*	0.2911(1)	0.288(2)	0.284(1)	0.287(1)
*y*	0.0472(1)	0.047(8)	0.047(1)	0.046(1)
*z*	0.2151(1)	0.211(6)	0.208(1)	0.208(1)
*U*_iso_ [Å^2^]	0	0.0120(10)	0.0003(14)	0.0013(16)
occupancy	1	1	1	1
O1
*x*	0.0636(8)	0.051(2)	0.065(1)	0.067(1)
*y*	0.3244(8)	0.331(1)	0.329(1)	0.325(1)
*z*	0.3476(9)	0.353(1)	0.345(1)	0.345(1)
*U*_iso_ [Å^2^]	0.19	0.017(02)	−0.0007(18)	0.0040(18)
occupancy	1	1	1	1
*x*	0.4402(8)	0.439(1)	0.440(1)	0.4376(6)
*y*	0.7546(11)	0.761(1)	0.760(1)	0.7586(6)
*z*	0.4782(9)	0.469(1)	0.4817(8)	0.4815(6)
*U*_iso_ [Å^2^]	0.03	0.0067(10)	0.0067(14)	0.0030 (16)
occupancy	1/0	1/0	1/0	0.64(5)/0.36(5)
*χ*^2^	–	–	1.575	1.413
*wR*_p_	–	0.1217	0.057	0.0539
*R*_p_	–	0.0898	0.0425	0.0404

**Figure 6:**
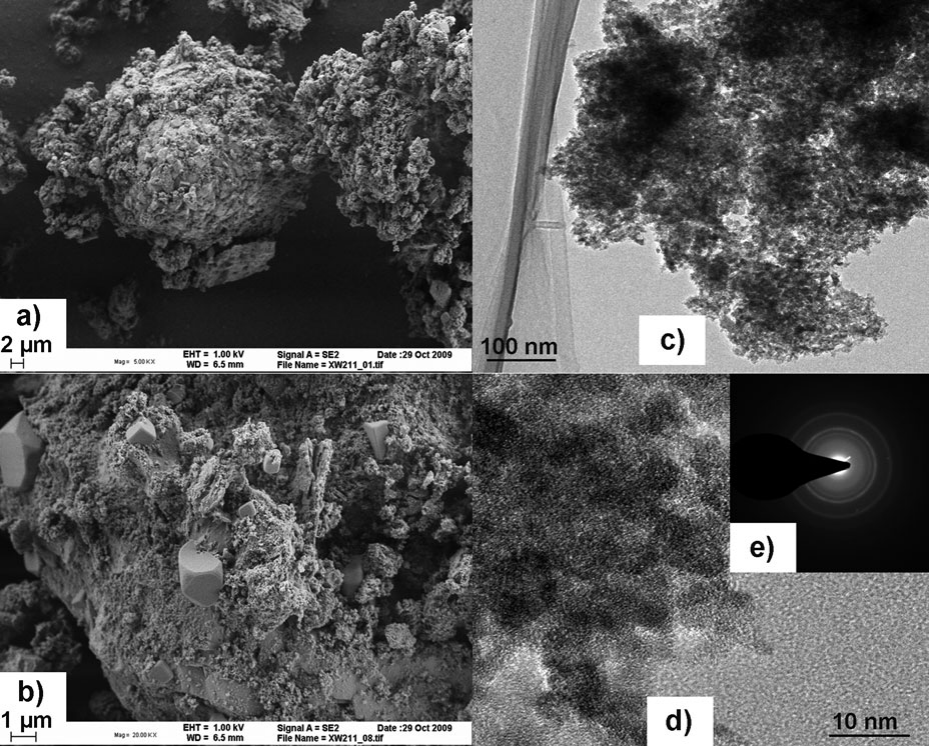
a, b) SEM and c, d) TEM images and e) ED pattern of sample 1.

**Figure 7:**
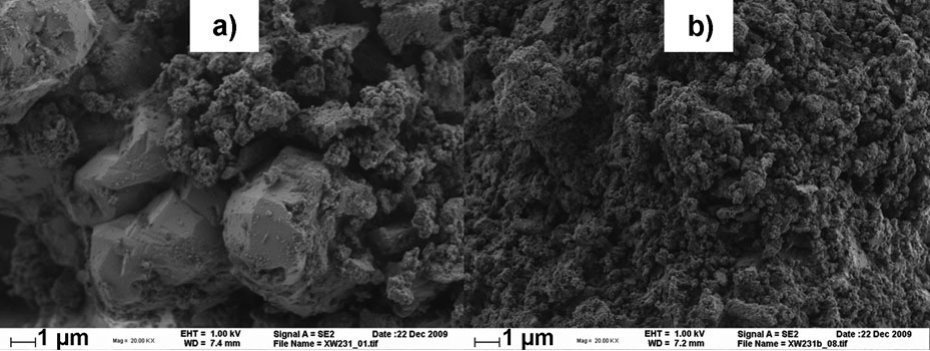
SEM images of a) pure sample 3 and b) carbon-coated sample 3.

**Figure 8:**
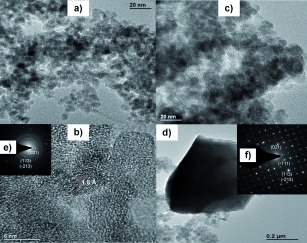
TEM images and ED patterns. a, b, e) Pure sample 3. c, d, f) Carbon-coated sample 3. Scale bars: a) 20 nm, b) 5 nm, c) 20nm, d) 0.2 μm.

It is known that fine powders are readily obtained by decomposition reactions of compounds, but at the same time, high temperature conversely drives these particles to stick and fuse together and thus is advantageous for the growth of single crystals. Figures 6–8 show SEM, TEM, and ED images of pure NbO_1.3(1)_N_0.7(1)_ and lactose-treated samples. The SEM images (Figure 6 a and b) reveal micrometric single crystals that are embedded in fine powders, the particle size of which is determined as 5–10 nm from TEM images (Figures 6 c and d, 8 a and b). Apparently, due to the existence of molten salt, single crystals grow larger when LiI is used as assistant flux (Figure 7). Moreover, the sample is clearly completely covered by carbon when NbO_1.3_N_0.7_ is treated with lactose (Figures 8 a and c). Samples coated with 4.6 wt % carbon have less distinct particle profiles than uncoated samples due to the carbon covering. However, it is difficult to differentiate whether the carbon really coats the original NbON particles or is just located between them. Remarkably, this addition of carbon indeed effectively improves the electrochemical properties, namely, the capacity and the cycling stability (see below). Besides, our results indicate that the nanoparticles and large single crystals both belong to the monoclinic NbON phase. First, the observable distance between atomic layers in a nanocrystal (Figure 7 b) of 1.8 Å corresponds to the (022) crystal plane in NbON. Furthermore, diffraction rings in the electron diffraction patterns indicate the multicrystalline nature of the selected area, which consists of nanometer-sized particles. By indexing the diffraction spots in the ED pattern of the large single crystal detected in TEM measurements, the phase was identified as monoclinic NbON (Figure 8 d, f).

Owing to the oxidation valence +4.7 of niobium in NbO_1.3_N_0.7_, the compound exhibits superparamagnetism (see Figure S1 of the Supporting Information). In fact, this phenomenon usually occurs when interparticle magnetic interactions are sufficiently weak in an assembly of nanoparticles.^14^ This evidence strongly supports the presence of nanometer-sized particles and the existence of an unsaturated oxidation state of niobium in these compounds.

**Figure 9:**
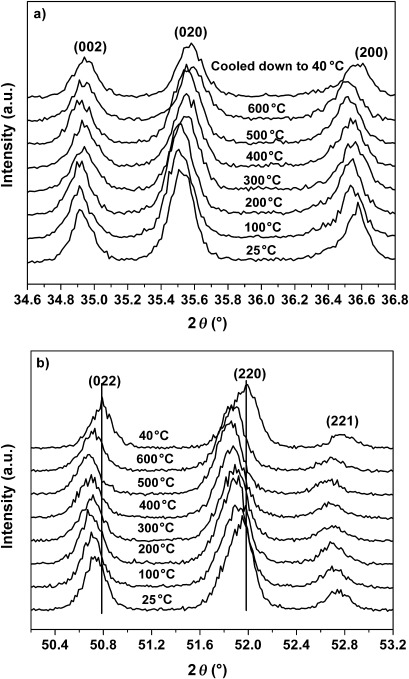
In situ temperature-dependent XRD patterns of NbO_1.3(1)_N_0.7(1)_. a) Angular range from 34.6 to 36.8°. b) Angular range from 50.2 to 53.2°.

**Thermal behavior**: The thermal stability of NbO_1.3(1)_N_0.7(1)_ was investigated by temperature-dependent XRD under vacuum. According to the XRD patterns in Figure 9, the lattice parameters of this oxynitride do not change much. For example, oxynitride perovskites AMO_2_N (A=Ba, Sr, Ca; M=Ta, Nb) exhibit even higher coefficients of thermal expansion than the corresponding isostructural oxides.^15^ Changes in octahedral tilting in perovskites contribute more to the thermal expansion coefficient than lattice volume expansion.^16^ However, in the monoclinic compound NbO_1.3_N_0.7_ investigated here, niobium is connected with seven O/N ions to form irregular octahedra. By comparison with symmetric and regular octahedra in perovskites, it becomes evident that tilting of [NbO_3_N_4_] polyhedra hardly affects the thermal expansion of compound, whereas extension of Nb—O/N bonds by heating apparently plays a more important role. Shilling et al. reported that baddeleyite- and fluorite-type oxynitrides always have lower volume thermal expansion coefficients than isostructural oxides.^17^ Hence, it is expected that monoclinic NbO_1.3(1)_N_0.7(1)_ would exhibit significantly low positive coefficients of thermal expansion. Shifting of the Bragg reflections which represent the [010] and the [001] zones after heating hints to a slight conversion of the compound. Compared with the initial compound, the sample after cooling to 40 °C has slightly shifted on reflections, which could originate from thermally induced changes in O or N content.

**Galvanostatic cycling**: The electrochemical performance of pure and lactose-treated NbO_1.3(1)_N_0.7(1)_ against Li was tested and compared under galvanostatic cycling conditions. During the annealing process the lactose is decomposed and gives a carbon coating of the oxynitride particles. Considering different lithiation mechanisms in different potential ranges, measurements were carried out at two potential cutoffs, 0.05 and 1 V. The first discharge was measured from open-circuit voltage (OCV) to final potentials and the subsequent charge cycled up to 3 V.

**Figure 10:**
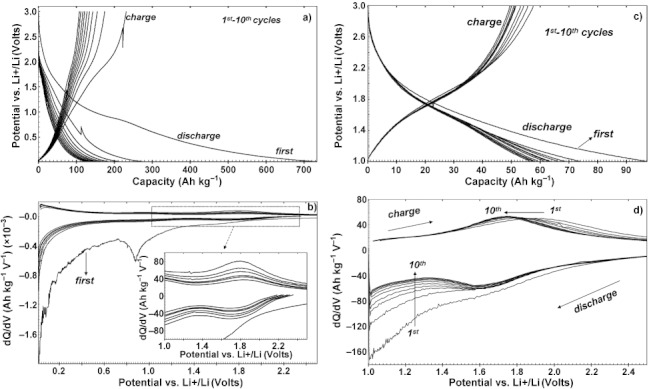
Voltage versus capacity profiles and corresponding differential capacity plots of pure NbO_1.3_(1)N_0.7_(1) in different voltage windows. a, b) 0.01–3 V; c, d) 1–3 V. All cycles were performed at a current density of 10 mA g^−1^.

**Figure 11:**
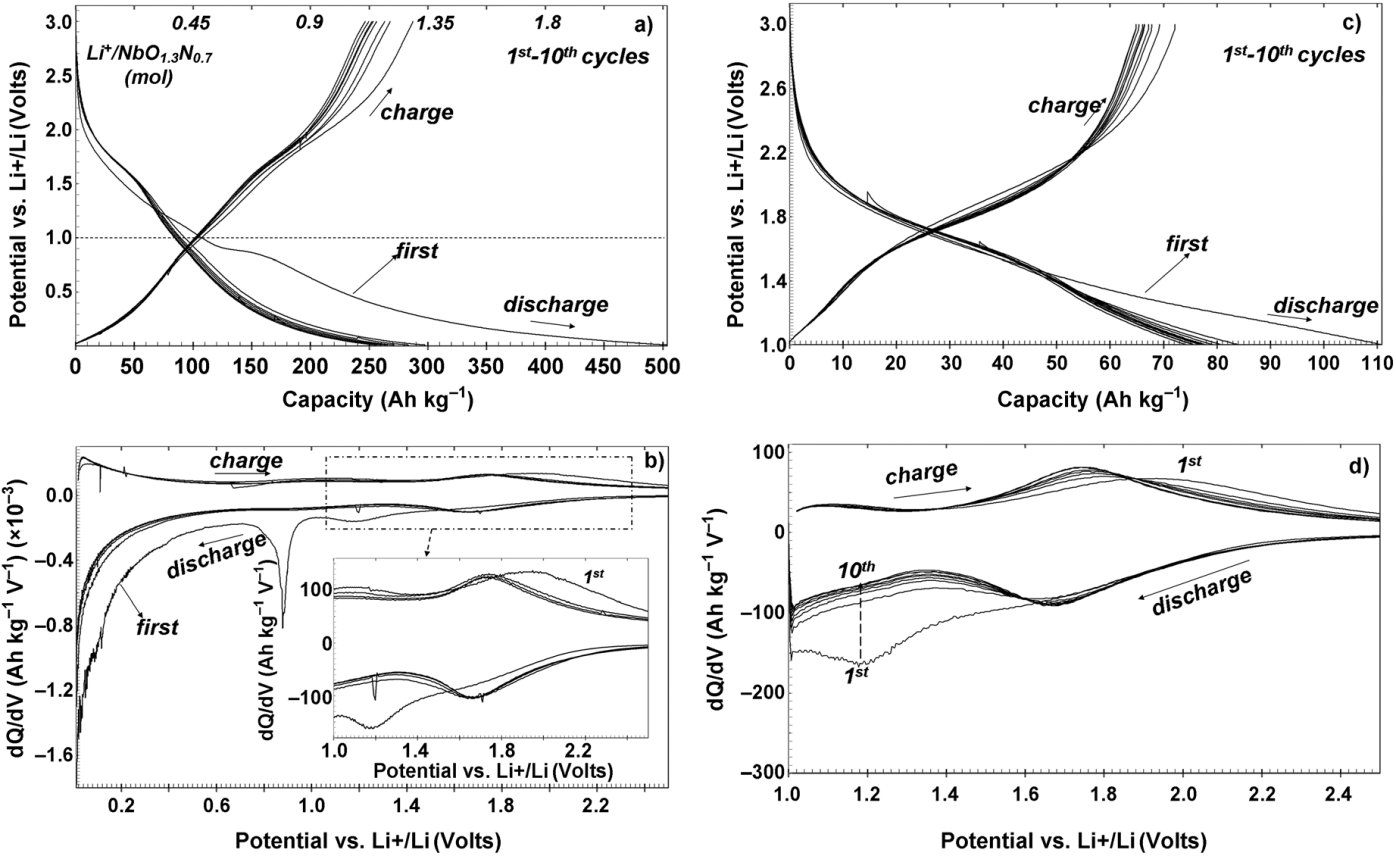
Voltage versus capacity profiles and corresponding differential capacity plots of NbO_1.3_(1)N_0.7_(1) with 4.3 wt % of carbon coating in different voltage windows. a, b) 0.01–3 V. c, d) 1–3 V. All the cycles were performed at a current density of 10 mA g^−1^.

When the potential ends at 0.05 V (Fig>ures 10 a and 11 a), the first discharge of NbO_1.3(1)_N_0.7(1)_ exhibits an extraordinarily high capacity of 500 to 700 A h kg^−1^, which afterwards drops rapidly to 200–300 A h kg^−1^ during subsequent cycles. Apparently, carbon-coated samples show better cycling performance than uncoated ones: the first discharge is followed by cycling with a quite stable capacity around 250 A h kg^−1^. The first voltage profiles of pure NbO_1.3(1)_N_0.7(1)_ show sloping plateaus at 0.9 V and below 0.5 V. In contrast, an extra 1.5 V plateau is observed for the carbon-coated sample. To investigate these potential peaks in detail, their differential capacity curves are plotted in Figures 10 b and 11 b, respectively. First, a sharp peak at 0.9 V occurs in the first discharge and then disappears in subsequent cycles. This plateau is frequently observed in electrochemical analysis of several molybdates, for instance, MnMoO_4_ and CaMoO_4_. The observed plateaus around 0.9 V are ascribed to breakdown of the metal oxide framework, catalytically enhanced by carbon.^18^ The largest contribution to the capacity occurs at 0.5–0.05 V due to insertion of lithium ions into the lattice with destruction of the crystal structure. Interestingly, this part of the process is reversible to a certain extent during the following charge and discharge cycles.

As the insets in Figures 10 b and 11 b show, reversible oxidation and reduction peaks around 1.7 V are observed during charge and discharge processes, which agree with the Nb^5+^/Nb^4+^ redox potential versus lithium. This behavior is similar to that of other niobates like Nb_2_O_5_, AlNbO_4_, and KNb_5_O_13_.^5^ For these compounds, further reduction of Nb^4+^/Nb^3+^ occurs at a potential plateau of 1.2 V. However, in our case, NbO_1.3(1)_N_0.7(1)_ only has an Nb^4+^/Nb^3+^ redox couple in the first cycle, which then disappears gradually. When the potential cutoff is set to 1.0 V, as shown in Figures 10 c and 11 c, these (de)lithiation processes become much more stable and reversible than in the potential range 3–0.05 V. Due to the improved electronic conductivity, a carbon-coated sample expectedly has better electrochemical behavior and still retains a capacity of 70–80 A h kg^−1^ in stable running. The oxidation peaks shift to smaller potential as charging continues, and simultaneously the peaks corresponding to the Nb^4+^/Nb^3+^ reduction start to fade. This may be explained by lithium insertion into various environments of the crystal structure. First, lithium is inserted into different voids formed by N/O anions during discharge, and then as more and more lithium remains in these specific positions, a different potential is needed to remove the rest of the lithium ions during charging.

On the basis of this discussion and previous studies on other similar niobates and molybdates, a charge–discharge reaction mechanism of NbO_1.3(1)_N_0.7(1)_ is proposed here. Basically, this process can be discussed in two potential regions (0–1 and 1–3 V), as indicated in Figure 11 a. For the first discharge, from OCV to 1 V, insertion of lithium is considered to occur by entry of Li^+^ into interstitial sites of the crystal structure of NbO_1.3_N_0.7_. A capacity as high as 110 A h kg^−1^ corresponds to intercalation of about 0.5 Li into the host [Eq. ].

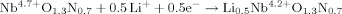
(5)

This reaction is reversible when cycling is continued in the range from 1 to 3 V. NbO_1.3(1)_N_0.7(1)_ and Nb_2_O_5_ have similar oxidation–reduction peaks, but there is a large difference in ability to insert lithium. No matter which structure Nb_2_O_5_ has, as much as 1.6–1.8 mol of Li^+^ can be inserted into 1 mol of Nb_2_O_5_. This can be ascribed to the existence of a number of easily traversed tunnels between edge- and corner-sharing [NbO_6_] octahedra. However, the space between [NbO_3_N_4_] units is irregular, small, and hardly fitting for lithium ions.

In the second voltage range, 1–0.05 V, the decreasing potential forces more lithium ions to diffuse into the compound, and breakdown of the host structure results. Such a conversion below 1.0 V occurs very frequently in oxide and nitride anodes.^2^ For these simple oxides or nitrides, it is easily understood that lithium would combine with oxygen or nitrogen to yield Li_2_O and Li_3_N, respectively. In the oxynitride studied here, we suppose that lithium preferably forms Li_2_O, and in parallel, Nb^4.7+^O_1.3_N_0.7_ is reduced to Nb^2.7+^O_0.3_N_0.7_. Obviously at room temperature, amorphous products appear after the conversion reaction. Consequently, after the conversion reaction powder XRD only shows broad angular distributions. Further lithium ions might be inserted into the reduced oxynitride NbO_0.3_N_0.7_ or another conversion reaction takes place.

In total, during the first discharge, 2.3 moles of Li^+^ enter into each mole of the oxynitride and Nb changes its valence from 4.7+ to 2.2+. However, the proposed reaction mechanism needs to be confirmed by further investigations.

## Conclusion

The oxynitride NbO_1.3(1)_N_0.7(1)_ with Ni^4.7+^ oxidation state was synthesized by decomposition of niobium (V) oxytrichloride ammoniate and chemically and electrochemically analyzed. Two methods were developed for preparation of samples: 1) direct decomposition of NbOCl_3_(NH_3_)_4_ and 2) an LiI-assisted method. In both cases, pure compounds were obtained and used to characterize the crystal structures, morphologies, and electrochemical performances against lithium. From elemental analysis and neutron diffraction, the compound was determined to be NbO_1.3(1)_N_0.7(1)_ instead of the expected NbON. The reaction products consist of 3–5 nm particles, but a few micrometric single crystals were found as well. NbO_1.3(1)_N_0.7(1)_ loses nitrogen on increasing temperature and transforms in air into Nb_2_O_5_ above 400 °C.

NbO_1.3(1)_N_0.7(1)_ was investigated with regard to its electrochemical performance in Li-ion batteries as an example of a lithium-free transition-metal oxynitride. Our study indicates that NbO_1.3(1)_N_0.7(1)_ coated with 4.6 wt % C is much more stable under reversible cycling than uncoated samples. When the cutoff potential is set to 0.05 and 1 V, respectively, the measured capacities reach 500 and 100 A⋅h kg^−1^ during the first discharge and then stabilize at 250 and 80 A⋅h kg^−1^ in subsequent cycling, respectively. A plausible mechanism has been proposed for the discharge–charge process of this oxynitride. This shows the potential of transition-metal oxynitrides as electrode materials of Li-ion batteries. Since the average potential of NbO_1.3(1)_N_0.7(1)_ is practically the same as that of Nb_2_O_5_, replacement of oxygen by nitrogen, which formally enhances the Li uptake capacity by 1 Li, does not necessarily lower the average electrochemical potential of the material. We believe that the increased charge of nitride anions compared to oxide anions is responsible for this observation. Clearly oxynitrides provide new opportunities as electrode materials in place of oxides.

## Experimental Section

The precursor compound NbOCl_3_ was prepared by gas-transport reaction. Nb_2_O_5_ (>99 %, JMC) and NbCl_5_ (99.8 %, Acros) were mixed in a molar ratio of 1:3, sealed in Pyrex tubes, heated to 400 °C over 4 h, and kept at this temperature for 40 h. This reaction yielded deep green needle-shaped crystals of NbOCl_3_. The compound was ground into a fine white powder and treated with ammonia at RT until the color changed into the bright yellowish of niobium(V) oxytrichloride ammoniate NbOCl_3_(NH_3_)_*x*_. To make sure the reaction was complete, a second grinding was applied. Because NbCl_5_ and NbOCl_3_ are both water-sensitive, all handling was carried out in a glove box under a dry Ar atmosphere.

NbON was synthesized by decomposition of niobium(V) oxytrichloride ammoniate. In a typical reaction, NbOCl_3_(NH_3_)_*x*_ (0.8 g) was sealed in a Pyrex tube (8 mm inner diameter and 1 m length), which was placed in a vertical tube furnace so that about 20 cm of the tube was outside of furnace. The temperature was set to 500 °C for 10–30 h. Black NbON powder was obtained at the bottom of the container and white NH_4_Cl condensed at the top of the Pyrex tube. If a flux salt was used, typically NbOCl_3_(NH_3_)_*x*_ (0.8 g) and LiI (1.7 g, ultra dry, 99 %, Alfa Aesar) were sealed in a Pyrex tube (8 mm inner diameter and 12 cm length), heated up 500 °C (100 K h^−1^) and kept at this temperature for 5–40 h in a muffle furnace. The black product was washed twice with deionized water, centrifuged, and dried at 100 °C for 2–3 h to give a final black powder of pure NbON.

Carbon-coated samples were prepared by mixing pure NbON and 15 wt % of lactose in water followed by subsequent drying at 100 °C. This product was transferred into a N_2_ flow furnace. The temperature was increased to 300 °C (10 K h^−1^) and then to 500 °C over 1 h, kept at 500 °C for 5 h, and cooled down as the furnace was switched off. 4.6 wt % of carbon was detected in the final sample.

Thermogravimetric (TG) and differential thermal analysis (DTA) were carried out on a Netzsch STA 409 C/CD instrument in argon atmosphere. For the study of the reaction, NbOCl_3_(NH_3_)_*x*_ (48.1 mg) was loaded in an alumina crucible The temperature was increased and decreased by 10 K min^−1^ from 20 to 1400 °C.

Powder X-ray diffraction (XRD) data were collected on a Bruker diffractometer (AXS mod. D8 Advance) with Bragg–Brentano geometry (*λ*_Cu__Kα1_
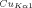
=1.54056 Å radiation (40 mA, 40 kV), germanium monochromator). The data for structure refinement were collected in steps of 0.015°, each 10 s over the 2*θ* range from 15 to 120°. Temperature-dependent XRD was carried out at steps of 0.015°, each for 0.2 s over the 2*θ* range from 5 to 90° while heating at a rate of 0.5 K s^−1^.

Neutron powder diffraction (NPD) data were collected with the PUS two-axis diffractometer at the JEEP-II reactor at Kjeller, Norway. A neutron wavelength of 1.5561 Å was used. The step size was 0.05 in the 2*θ* range from 10 to 135°. The sample was contained in a vanadium can. Rietveld refinements for XRD and NPD were both performed with the GSAS software.^19^

The carbon content of the lactose-treated sample was analyzed in Laboratorium für Organische Chemie (ETH Zürich). The O/N content of the samples was measured by the hot-gas extraction method by using a LECO TC500 analyzer at EMPA (Dübendorf, Switzerland).

Scanning electron microscopy (SEM) analysis was carried out on a Zeiss Gemini 1530 operated at 1 kV. For transmission electron microscopy on a CM30ST (FEI; LaB_6_ cathode) and a Tecnai F30 microscope (both operated at 300 kV, point resolution≍2 Å) the material was deposited onto a holey carbon foil supported on a copper grid.

A lithium metal (0.75 mm-thick ribbon, Aldrich) was taken as anode and Merck Selectipur LP30, which consists of 1 m solution of LiPF_6_ in a mixture of ethylene carbonate and dimethyl carbonate (1:1 w/w) as electrolyte. Electrodes of NbON/Super P carbon/PVDF (80:10:10 wt %) were ground and then ultrasonically dispersed in *N*-methyl-2-pyrrolidone (NMP) for 30 min at 40 °C. The obtained slurry was printed on titanium current collectors from a dropper, and then the solvent was evaporated at 150 °C for 1 h and 100 °C overnight. The typical weight of such electrodes is about 5 mg. Galvanostatic discharge/charge curves were measured with an applied current of 10 A h kg^−1^.
