# Supplementary material for: Niobium(V) Oxynitride: Synthesis, Characterization, and Feasibility as Anode Material for Rechargeable Lithium-Ion Batteries
Source: Chemistry. 2012 Mar 29;18(19):5970–8. doi: 10.1002/chem.201102653 (PMC3528069; doi:10.1002/chem.201102653)
Supplement: Supplementary file 1 [file chem0018-5970-sd1.pdf]

CHEMISTRY
A EUROPEAN JOURNAL

Supporting Information

© Copyright Wiley-VCH Verlag GmbH & Co. KGaA, 69451 Weinheim, 2012

Niobium(V) Oxynitride: Synthesis, Characterization, and Feasibility as Anode Material for Rechargeable Lithium-Ion Batteries

Xiao-Jun Wang,*[a] Frank Krumeich,[a] Michael Wörle,[a] Reinhard Nesper,*[a] Laurent Jantsky,[b] and Helmer Fjellvåg[b]

chem_201102653_sm_miscellaneous_information.pdf

Magnetic Measurements

The measurements of magnetism were made using a Quantum Design SQUID (Superconducting Quantum Interference Device) magnetometer MPMS 5S. The applicable field is in the range of +-5T and temperature 1.7-400K. Sample No.3 (see table 1) was measured in a T-loop, changing temperature from 2K - 300K - 2K at constant external field at 50, 1000, and 5000 Oe. Firstly sample was cooled down to 2K at zero field (ZFC), then the field was set and measured while warming. Then the temperature decreased to 2K and kept the field at the same level (FC).

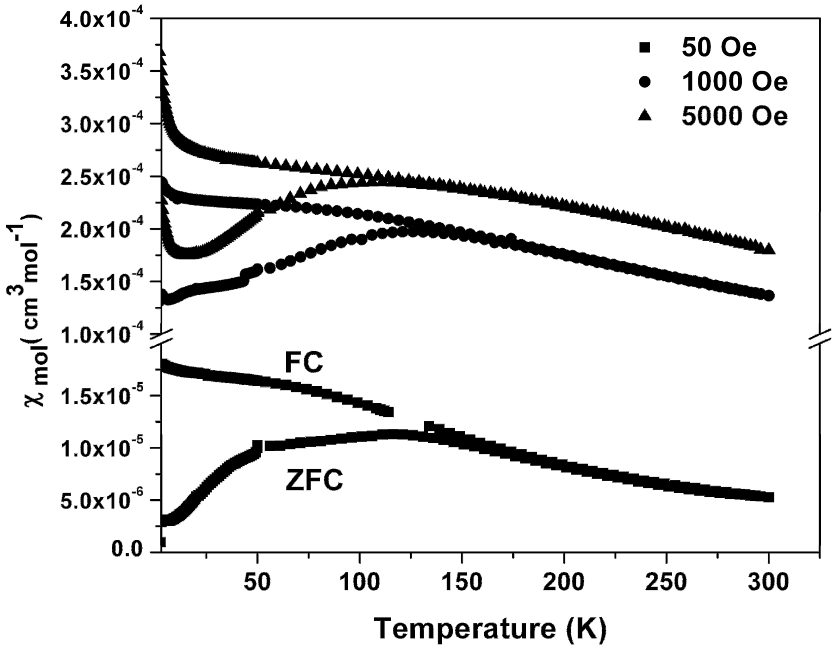

Figure S1. Temperature dependence of magnetic susceptibility of nanosized $NbO_{1.3(1)}N_{0.7(1)}$ measured under different fields
